# Properties, Antioxidant and Antibacterial Activity of Southern Meagre Fish (*Argyrosomus hololepidotus*) Skin Gelatin Reinforced with Clove Bud Extract

**DOI:** 10.3390/gels11010021

**Published:** 2025-01-01

**Authors:** Parvin Rostami, Ali Taheri, Mostafa Ghaffari

**Affiliations:** Fisheries Department, Faculty of Marine Sciences, Chabahar Maritime University, Chabahar 9971778631, Iran; parvinerostami91@gmail.com (P.R.); mgmostafaghaffari@gmail.com (M.G.)

**Keywords:** film, fish gelatin, clove bud extract, antibacterial, antioxidant

## Abstract

The properties of biopolymer films prepared using Southern meagre fish (*Argyrosomus hololepidotus*) skin gelatin blends, both with and without clove bud extract (CE) at concentrations of 0.3% and 0.7%, were investigated. The addition of CE enhanced the light barrier properties and decreased water vapor permeability from 1.68 to 0.85 (×10^−13^ g s^−1^m^−1^Pa^−1^) (*p* < 0.05) in the films that contained CE. Additionally, the films’ water solubility diminished as the concentration of CE increased (89.20 to 69.04%) (*p* < 0.05). SEM images revealed a smooth, uniform surface without cracks in the samples both with and without CE, whereas the films that included CE displayed a rougher and denser cross-section. FTIR spectra revealed variations in peaks between the films containing CE and those without it. The incorporation of CE raised the glass transition temperature (51.04 to 58.80 °C) and the melting temperature (124.65 to 141.92 °C) of the films. Additionally, the antioxidant activities, assessed through DPPH free radical scavenging activity (86.97%) and reduction power (λ of 0.85), along with moderate antibacterial activities against four distinct foodborne pathogens, improved with increased concentrations of CE. It can be concluded that phenolic compounds, such as eugenol in the clove extract, facilitated the formation of additional bonds between the peptide helixes of the gelatin, thereby enhancing the properties of the CE-incorporated films. Thus, Southern meagre fish gelatin film containing CE is an effective active packaging biomaterial for seafood products, exhibiting satisfactory properties.

## 1. Introduction

In the last ten years, health and environmental concerns regarding synthetic petroleum-based packaging used in the seafood industry have become a major problem for food safety. Recently, to enhance the shelf life of seafood products, active packaging using bio-based biodegradable edible films has been considered a significant concept in the food industry. The research on edible films for seafood industries is necessary because, currently, less than 10% of petroleum-based plastic waste is degraded globally, and much of the remaining waste turns into microplastics, negatively affecting human health and ocean fauna [1,2]. Edible films possess good properties of practicality, biodegradability, and safety, which enhance their potential for seafood packaging applications [3]. Edible films are produced from natural polymers, such as proteins, starch, lipids, cellulose, and other polysaccharides [4,5,6].

Gelatin, as a bio-based biopolymer, has garnered considerable interest in the field of edible film research because of its remarkable properties, including biodegradability, biocompatibility, film-forming characteristics, and barrier properties against volatile compounds, UV light, gases, and oils. It also has a low taste and aroma, stability, and commercial availability [7,8,9,10]. Over 90% of commercial gelatin is produced from porcine and bovine collagen globally [7,11]. However, certain health concerns, governmental restrictions in some European countries, and religious restrictions for kosher and Muslim diets complicate the use of mammalian gelatin. Consequently, alternative sources for gelatin extraction, such as marine animals—especially marine fish—are increasingly being considered [11,12].

Active agent-containing edible films can improve food quality by preventing bacterial proliferation and minimizing lipid oxidation [13]. Fish gelatin films can be enriched with plant extracts that contain bioactive compounds to improve the functional characteristics of the products, including their antioxidant and antibacterial properties [14]. According to the literature, various gelatin films have been developed by incorporating plant extracts, such as clove extract, into gelatin. The resulting films demonstrated effective antibacterial and antioxidant properties [15,16,17,18,19]. Additionally, films containing clove extract exhibited better barrier properties compared to the control [20].

Clove (*Syzygium aromaticum*) is a traditional medicinal plant used to treat nausea, stomach diseases, colon diseases, vomiting, malaria, candida, viral infections, cholera, tuberculosis, and foodborne infections in Asia (India, China, Indonesia, and Iran) and America [21]. The methanol extract of clove bud powder demonstrated a high DPPH free radical scavenging capacity (92.2% at a concentration of 200 µL) and antimicrobial properties (inhibition zone of 17.33–19.33 mm at a concentration of 25 µL). Phytochemical investigations reveal that eugenol, a member of the phenylpropanoid class of compounds, along with its derivatives, contributes to the distinctive aroma of *Syzygium aromaticum*. These compounds exhibit a range of beneficial properties, including antimicrobial, insecticidal, antioxidant, antitumor, anesthetic, and anti-inflammatory effects. Among the phenolic compounds identified in the clove bud extract, eugenol acetate, ferulic acid, flavonoids, and caffeic acid were highlighted, with eugenol (2-methoxy-4-(2-propenyl) phenol) being the predominant component, accounting for 19.46% of the extract [22,23,24,25]. Furthermore, eugenol is highly valued in dentistry due to its ability to penetrate dental pulp. The high level of eugenol in clove bud extract contributes to its antioxidant, antibacterial, and health-promoting properties [26].

In addition, meagre fish (*Argyrosomus hololepidotus*), belonging to the Sciaenidae family (drums or croakers), is a delicious fish from the Oman Sea that is filleted in Iran (domestic name: Mish Mahi). Therefore, the skin of this fishery product can serve as a valuable source for gelatin production.

Given its antioxidant properties and safety profile, clove bud extract can be used as a natural antioxidant in packaging, especially in films made from gelatin. In this context, the objective of the current study is to develop a degradable meagre skin gelatin film containing clove bud extract for seafood packaging in order to extend the shelf life of the seafood products. As a result, the physical, chemical, and technological characteristics of fish gelatin coatings that include clove bud extract will be examined.

## 2. Results and Discussion

### 2.1. Film Thickness and Water Solubility

The characteristics of the film are affected by its thickness. The film’s thickness is affected by plasticizers, biopolymer content, emulsifiers, and the active ingredients of the extract [27].

All produced films were uniform and had smooth surfaces. The obtained films had a small thickness (0.24–0.26 mm, *p* > 0.05) and were easily separated from the plate surfaces. The hydrophobicity of the films increased, and the moisture content was decreased by adding CE at concentrations of 0.3% and 0.7%. The thickness of the films did not exhibit any notable differences; however, the control film had the highest moisture content value, which can be attributed to the hydrophilic properties of gelatin and glycerol. The addition of CE led to a reduction in the moisture content of the films, similar to the findings observed for gelatin incorporated with palm oil [28] and gelatin/guar gum/grape seed oil [29]. In addition, in some different studies, no significant differences in the thicknesses of the cuttlefish skin gelatin/herbal extract (including clove extract), gelatin/green tea extract, and gelatin/green tea extract incorporated with lemon essential oil films were observed [30,31,32], which aligns with the results of the current study.

The control film showed the maximum water solubility. In contrast, films incorporated with 0.3% and 0.7% clove extract demonstrated reduced water solubility. Notably, the 0.7% clove extract film showed a significant difference in water solubility compared to the control film (*p* < 0.05). Nevertheless, there were no notable differences found between the 0.3% clove extract films and both the control and 0.7% clove extract films (*p* > 0.05). Water solubility is a crucial attribute of edible films, significantly influencing the shelf life of food products. Changes in glycerol and the dry matter concentration of the film influence its solubility in water [33]. The decrease in the solubility of the film may be attributed to the hydrophobic nature of the clove bud extract. When clove bud extract is added to the film, it reduces the solubility due to crosslinking. Phenolic compounds from the herbal extracts interact with the protein helix through hydrophobic interactions, leading to a stronger film network structure and decreased water solubility [34]. Bio-based films used in food packaging must have good water-resistant properties, especially in humid conditions [35].

In a study by Wu et al. [31], which examined gelatin extracted from silver carp and the preparation of films using green tea extract, the results indicated that solubility decreased as the amount of green tea extract increased. They suggested that the low solubility in films containing green tea extract might be attributed to enhanced crosslinking. Furthermore, Hoque et al. [30] found that adding clove extract to cuttlefish skin gelatin decreased the film’s solubility in comparison to the control (*p* < 0.05). The control gelatin film, characterized by a high content of hydroxyl groups, possessed excellent hydrophilic properties [36]. In comparison, films incorporating herbal phenolic extracts exhibited reduced water solubility, making them more suitable for practical applications.

### 2.2. Water Vapor Permeability

The results are presented in Table 1. In the film with 0.7% clove extract, the water vapor permeability was lower than that of the control and the 0.3% clove extract film (*p* < 0.05). Meanwhile, the film with 0.3% clove extract did not exhibit a significant difference from the control (*p* > 0.05).

Overall, a lower water vapor permeability (WVP) minimizes the transfer of vapor from the environment to the product, thereby improving the product’s freshness [37].

The movement of water between the food item and its surrounding environment affects the shelf life of the products, as spoilage is directly related to the moisture content within the packaging. As a result, packaging materials, like film products, need to reduce moisture transfer to enhance the shelf life of the products [38,39]. The hydrophilic nature of gelatin, as a result of its polar amino acid and hydroxyl side chains (-OH), can enhance water permeability of gelatin films [29,30]. In contrast, incorporating gelatin films with extracts or oils that increase hydrophobic interactions and hydrogen bonds can reduce the WVP of the gelatin film. The literature indicates that herbal extracts can play a crucial role in this regard. For instance, the incorporation of grape seed oil into the gelatin/guar gum film significantly reduced WVP values (*p* < 0.05) [29]. Comparable findings were noted with murta leaf extracts added to gelatin films made from tuna fish (Thunnus thynnus) [40], seaweed extract/gelatin film [41], and cinnamon, clove, and star anise extracts incorporated into the gelatin film of cuttlefish skin [30], as well as the ethanolic extract of coconut husk incorporated into tilapia skin gelatin film [42].

In the present study, the film containing 0.7% clove extract (CE) showed the lowest water vapor permeability (WVP) value (*p* < 0.05). The interaction between the protein molecules in gelatin and the phenolic compounds in clove extract may decrease the presence of charged or polar residues in the gelatin. This reduction could lead to decreased water adsorption, subsequently lowering the WVP of the films [43]. The polyphenolic compounds in clove extract could also reduce water vapor permeability by promoting crosslinking through hydrogen bonds or hydrophobic intercommunications with the gelatin matrix. An increase in crosslinking can reduce the free volume of the matrix. The degree of crosslinking and the density of the free volume in the film matrix play a significant role in influencing these properties [31,44].

### 2.3. Light Transmission and Transparency

The light transmission across the wavelength spectrum of 200–800 nm for all three types of films (control, 0.3%, and 0.7% clove extract) is presented in Table 2. The film enriched with clove extract (CE) exhibited a lower transmission percentage compared to the control film at wavelengths of 200–280 nm (UV). Light transmittance at wavelengths of 350 to 800 nm was also lower in the CE enriched gelatin film compared to the control. The control film was very transparent, while the addition of clove extract increased the turbidity in the fish gelatin film (*p* < 0.05).

Decreases in light transmission were observed for both the 0.3% and 0.7% clove extract (CE) films at all wavelengths compared to the control film. The transmission for 200 and 280 nm (UV light) for both CE incorporated films and control was low, with the 0.7% CE film exhibiting the lowest transmission. According to the literature, gelatin films possess high barrier properties against UV light [30,45]. The presence of aromatic amino acids in gelatin may be responsible for the absorption of UV light [46].

Additionally, the film incorporated with 0.7% CE (*w*/*w*) demonstrated the lowest transmission in the visible range of 350–800 nm. This may be attributed to the formation of gelatin–phenolic complexes, which enhance light scattering as it passes through the film [42]. Overall, incorporating clove bud extracts into gelatin films significantly affected the transmission of visible light and increased the transparency values of the films (*p* < 0.05). A rise in the transparency value signifies a reduction in the clarity of the incorporated films. Previous studies have shown that the transparency of films tends to decline when extracts are added [42,47,48]. Several factors influence the transparency of gelatin-based films, including film thickness, production conditions, and the interactions between gelatin amino acids and herbal extracts [30,42,49,50,51]. Additionally, incorporating a higher concentration of herbal extracts can lead to the aggregation of protein molecules, which in turn increases the turbidity of the film [42,52].

### 2.4. Microstructure of the Films

**Figure 1:**
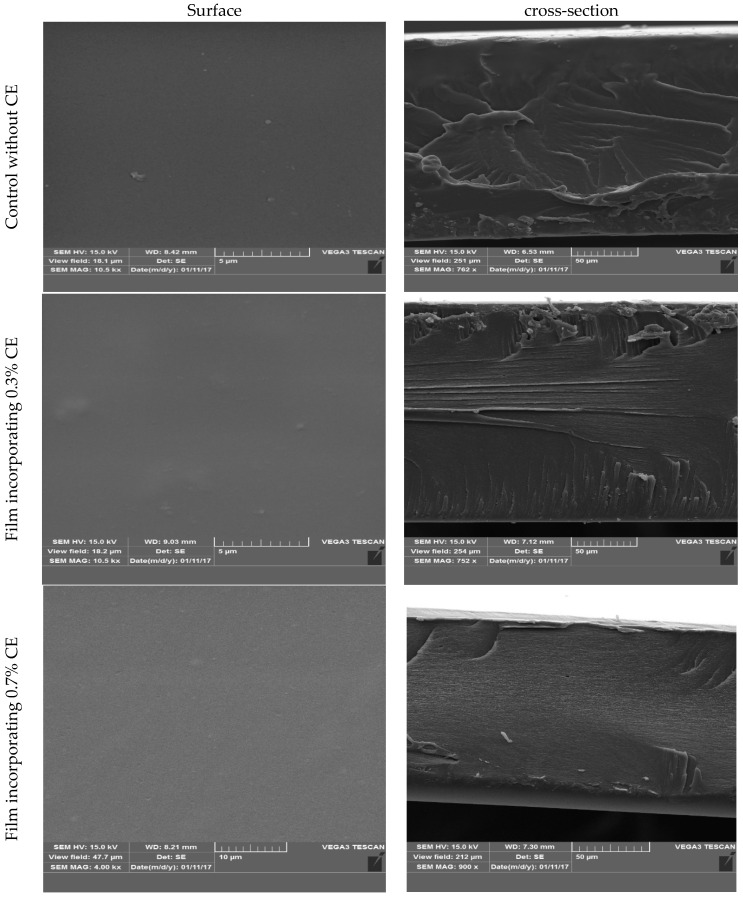
Scanning electron microscope images of the surface and cross-section of the films containing glycerol as the control (**above**), 0.3% clove bud extract (**middle**), and 0.7% clove bud extract (**lower**).

To investigate the microstructural characteristics of the produced biopolymers, the films’ surface and freeze-fractured cross-section were examined with a scanning electron microscope. Figure 1 presents micrographs of the control film, as well as the films containing 0.3% and 0.7% clove extract. The images reveal that all three types of films have a smooth, homogeneous surface free of cracks, which is the surface morphology specification of fish gelatin films documented by Nilsuwan et al. [53]. The smooth and homogeneous surface of the films indicates a well-groomed film matrix.

The cross-sectional area of the control film displays a smooth and dense structure, free from any granular or porous characteristics. The findings regarding cuttlefish gelatin films containing extracts of star anise, clove, cinnamon, or tilapia gelatin incorporated with coconut husk extract and fish gelatin films combined with epigallocatechin gallate resembled the results of this study [30,42,48]. In addition, the films containing the extract display a rougher and more condensed cross-section. The compactness and water vapor barrier properties could be enhanced by interactions between phenolic compounds and gelatin peptides [42].

SEM serves as a highly effective technique for investigating the consistency and microstructure of active films made from gelatin. The incorporation of hydrophobic compounds and plasticizers can influence the interactions between protein chains, subsequently altering the physical characteristics of the products. Consequently, the microstructure of biodegradable films has a direct impact on their physicochemical and barrier properties [9,54,55]. Enhanced interactions between proteins and polyphenols in gelatin and CE may lead to the development of a film with a denser structure. The roughness noted may result from the transformation of phenol into quinone, which facilitates considerable aggregation within the film’s structure via non-covalent bonds [30].

### 2.5. Fourier Transform Infrared (FTIR) Spectroscopy

**Figure 2:**
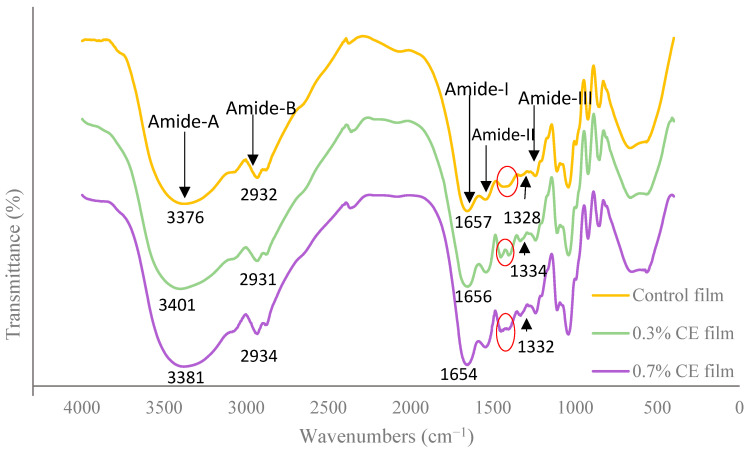
FTIR spectra of control, 0.3% clove bud extract, and 0.7% clove bud extract films.

The FTIR spectroscopy results for the control, 0.3% CE, and 0.7% CE films are presented in Figure 2. The FTIR analysis reveals that all three film types (control, 0.3% CE, and 0.7% CE) display similar spectra, indicating that no significant changes have occurred in the primary groups of the gelatin films. In the control, 0.3% CE, and 0.7% CE films, the bands at 3376.82, 3401.08, and 3381.94 cm⁻^1^ are associated with amide A. Amide A refers to the NH stretching that is associated with hydrogen bonding, reflecting the degree of transverse intersection [29]. The rise in absorption for amide A may be linked to the stretching of OH groups in the phenolic components of clove extract [56]. In films containing clove extract, the amide A peak appears at a higher frequency, suggesting an enhancement in the transverse intersection due to the incorporation of the extract.

For the control, 0.3% CE, and 0.7% CE films, the amide B bond was detected at 2932.63, 2931.58, and 2934.96 cm⁻^1^, respectively. This is associated with CH antisymmetric and symmetric stretching, as well as –NH_3_+ bands [19]. Additionally, a second peak at 2880 cm⁻^1^ corresponds to C–H stretching in CH_2_ groups [29]. The vibration of amide I was identified at 1657.21, 1656.06, and 1654.80 cm⁻^1^, respectively. This vibration is related to the C=O stretching and hydrogen bonding coupled with COO [19].

The vibration of amide II in the control, 0.3% CE, and 0.7% CE films was detected at 1544.05, 1540.32, and 1546.13 cm⁻^1^, respectively. This vibration is linked to the NH bending coupled with the CN stretching [57]. The frequency of amide II was lower in the 0.3% CE film compared to the control film, while it was higher in the 0.7% CE film than in the control.

The variations observed in amide I and II suggest that the incorporation of the extract may influence the structure of the α-helix or modify the secondary structure of the peptide in the resulting film.

Amide III was identified at 1243.70, 1241.87, and 1241.46 cm⁻^1^ in the control, 0.3% CE, and 0.7% CE films, respectively. This amide is linked to the breakdown of the collagen triple structure and the transformation of the α-helix into a random structure, as a result of gelatin formation. Amide III reflects vibrations of C-N and NH in the amide bond, as well as CH_2_ in glycerol or the CH_2_ groups of glycine [58]. A vibration was recorded at 1328.67 cm⁻^1^ in the control film, and this vibration was also observed in the 0.3% and 0.7% CE films at 1334.09 and 1332.25 cm⁻^1^, respectively. This vibration is related to derivatives of the proline amino acid and signifies the establishment of bonds between peptide carboxyl ions and the chemical ions that have been incorporated [59].

The bands at 1270 cm⁻^1^ and 1240 cm⁻^1^ correspond to C–O stretching vibration of phenolic hydroxyl groups [60]. The absorption intensity in these two bands was greater in the 0.7% CE film, and both films containing clove extract exhibited higher absorption intensities than the control film. This suggests a successful integration of clove bud extract into the fish gelatin structure. The bands at 1041.41, 1040.48, and 1041.83 cm⁻^1^ are associated with the OH group of glycerol. In the 0.3% CE film, two bands were noted at 1404.06 and 1452.27 cm⁻^1^, while the 0.7% CE film showed these bands at 1401.39 and 1451.32 cm⁻^1^. In contrast, the control film displayed only one band at 1437.58 cm⁻^1^, as highlighted by the red circle in Figure 1. The presence of bands in the range of 1451–1452 cm⁻^1^ indicates C–C stretching of the aromatic rings of eugenol found in the clove extract [61]. It can be concluded that eugenol in the clove extract has facilitated the formation of additional bonds at these frequencies, which were not present in the control film due to the absence of this compound.

### 2.6. Differential Scanning Calorimetry

**Figure 3:**
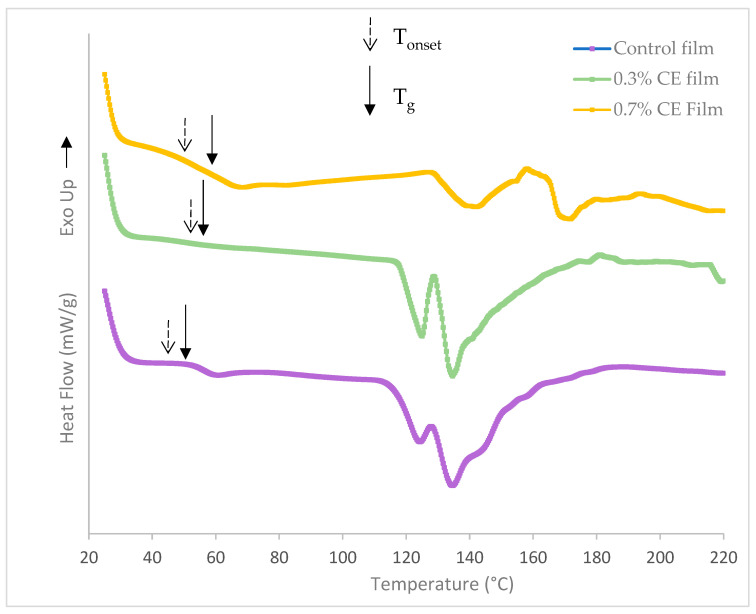
DSC thermogram of the control, 0.3% clove bud extract, and 0.7% clove bud extract films.

The results of gelatin films with or without CE showed in Figure 3 and Table 3, which show the glass transition temperature (T_g_), onset temperature (T_0_), and melting temperature (T_m_).

The endothermic peak observed in the range of 40–70 °C in marine gelatin films is primarily attributed to the glass-to-rubber transition of gelatin [62]. The control film exhibited the lowest T_g_ at 51.04 °C. In contrast, the incorporation of 0.3% and 0.7% clove bud extract into the gelatin film resulted in higher T_g_ values of 57.12 °C and 58.80 °C, respectively. This suggests that the addition of clove extract (CE) up to 0.7% into fish gelatin can help maintain the integrity of the resulting biocomposite. Consistent with the findings of this study, Nilsuwan et al. [48] reported an increase in the glass transition temperature (T_g_) of fish gelatin films, rising from 45.54 °C for the control film to 51.86 °C with the incorporation of epigallocatechin gallate at a concentration of 0.2%. Additionally, Nagarajan et al. [42] found that the control tilapia gelatin film exhibited the lowest T_g_ at 47.14 °C. In contrast, the addition of ethanolic extract from coconut husk to the control gelatin film resulted in higher T_g_ values of 54.99 °C and 53.57 °C, respectively. The glass transition refers to the movement of molecular segments within the disordered (amorphous) structure, shifting from a fragile glassy solid to a more flexible rubbery state. On the other hand, the melting transition of the protein film signifies the temperature at which the organized or aggregated structure begins to break down [63].

T_g_ is a crucial parameter that impacted the properties and water vapor permeability of the biodegradable films [64]. Generally, the DSC results confirmed the water vapor barrier properties of the films (Table 1). Adding higher concentrations of clove extract (CE) can lead to crosslinking in the gelatin matrix, which enhances the strength and stability of the gelatin film network when exposed to heat. The observed increase in the glass transition temperature (T_g_) of these films can be attributed to the interactions between hydrogen bonds or hydroxyl groups present in gelatin peptides and phenolic compounds, such as eugenol (2-methoxy-4-(2-propenyl) phenol) [42]. Studies have shown that the inclusion of phenolic compounds enhances protein interactions, reduces the mobility of gelatin chains, and reinforces the cohesive structural integrity and the crosslinking of the biopolymers within the films [65].

Two endothermic melting transition were observed alongside the T_g_ transition, specifically at temperatures of 124.19–141.92 °C and 134.20–171.38 °C. The temperatures of the transition in the control and 0.3% CE films were lower than those were in the 0.7% CE film. In this context, Mutlu [29] reported that the incorporation of grape seed oil into the gelatin/guar gum film matrix resulted in a broader and higher T_max_. The endothermic change that takes place following the glass transition is probably linked to the helix–coil transition of gelatin [66]. Furthermore, the disruption of connections within the film samples’ structures might have played a role in the melting transition. During the extended drying period of the cast film, gelatin chains may experience renaturation, resulting in the development of a more structured arrangement [67].

Incorporating natural extracts into the films necessitates greater enthalpy to break the inter-chain interactions, leading to a higher melting point than that of pure gelatin films. This can be explained by the creation of crosslinking, including hydrogen bonds or hydrophobic interactions between the natural extracts and the reactive groups of polypeptides in gelatin [68]. These findings align with the results obtained from FTIR analysis, suggesting that clove extract may act as a heat barrier by reinforcing the film network. Consequently, the endothermic transition associated with the decomposition of the gelatin matrix occur at higher temperatures in the 0.7% CE film.

### 2.7. Bioactivity of the Films

#### 2.7.1. Antioxidant Properties of the Films

**Figure 4:**
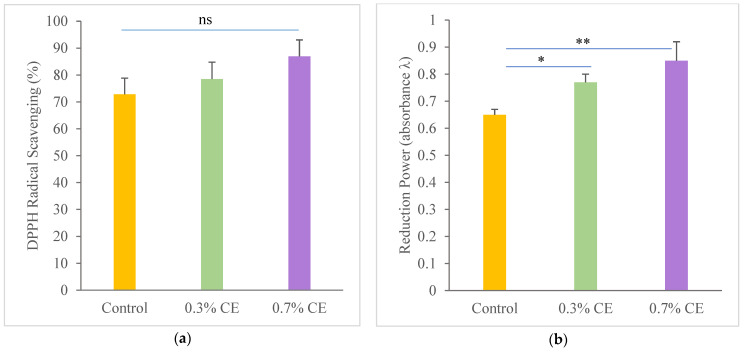
DPPH radical scavenging capacity (**a**) and reduction power (**b**) of the fish gelatin films without and with incorporated clove bud extract. ns: not significant; *: *p* < 0.05, **: *p* < 0.001. ns: non-significant.

Figure 4a displays the results of the free radical scavenging activity of the films produced. All films demonstrated effective free radical scavenging activity, exceeding 69%. The films containing 0.3% and 0.7% CE exhibited no significant differences when compared to the control film (*p* > 0.05). However, numerically, the control film exhibited the lowest amount of scavenging activity. In contrast, the films containing 0.3% and 0.7% clove extract demonstrated an increase in scavenging activity. Furthermore, Figure 4b illustrates the reduction power of the produced films. The control film exhibited the least reduction power. In contrast, the film with 0.3% CE demonstrated a significant difference from the control (*p* < 0.05), while the film containing 0.7% CE showed the highest reduction power, significantly differing from the control (*p* < 0.001).

The DPPH free radical scavenging assay is among the most effective techniques for assessing the antioxidant activity of herbal extracts [69]. The incorporation of clove extract at concentrations of 0.3% and 0.7% increased the free radical scavenging activity in the films, rising from 72.82% to 86.95%. Clove is recognized as an excellent antioxidant agent [18]. Furthermore, clove bud extract has demonstrated notable antioxidant properties across various meat products, indicating its potential as a natural preservative [70]. The principal active compound in clove extract is eugenol, a phenolic compound recognized for its extensive range of biological activities, including antimicrobial and antioxidant effects [16]. Phenolic compounds, characterized by the presence of hydroxyl groups (-OH), play a crucial role in antioxidant mechanisms. These hydroxyl groups can donate electrons to free radicals, effectively neutralizing them and preventing the oxidation of other sensitive components within seafood matrices [71]. This electron donation not only helps to maintain the quality and shelf life of meat products but also contributes to the overall health benefits associated with the consumption of clove bud extract. The incorporation of such natural antioxidants in food preservation strategies aligns with the growing demand for clean-label products and the reduction of synthetic additives in the seafood industry.

The antioxidant activity of herbal extracts is not exclusively due to phenolic compounds; other substances, like terpene alcohols, aldehydes, ketones, and ethers also play a role in free radical absorption of specific extracts [72,73]. Additionally, interactions between phenolic agents and amino acids can improve the antioxidant properties of the products [74].

The lack of significant difference between the control and CE films in DPPH scavenging activity may be attributed to the concentrations used in this study. Higher concentrations might yield different results compared to the control. Ji et al. [55] found that at low concentrations of cinnamaldehyde (CA) in the gelatin/CA film, there was no notable difference in antioxidant activity compared to the control film. However, with higher concentrations of CA, the antioxidant activity of the active films showed a significant improvement (*p* ≤ 0.05).

In the present study, the control film demonstrated notable antioxidant capacity. In contrast, some other research has reported negligible antioxidant activity for gelatin without the incorporation of an antioxidant agent. Nevertheless, Gómez-Guillén et al. [40] discovered that films produced from tuna skin gelatin demonstrated strong antioxidant activity. Fish gelatin naturally possesses antioxidant activity due to several amino acids, such as glycine and proline, which are present in the peptide bonds formed during film production [73]. The –NH_2_ or –OH groups found in the side chains of gelatin peptides might also contribute to their radical absorption properties [75]. Therefore, the bioactive peptides in Southern meagre fish gelatin likely have potential antioxidant activity, which may enhance the antioxidant properties of the control film.

Some studies have shown that the antioxidant properties of edible films can be enhanced by incorporating phenolic or other active compounds. Examples include tilapia skin gelatin films with bergamot essential oil and lemon essential oil [47], gelatin with aloe vera extract [76], starch with clove bud essential oil [77], gelatin with mangrove (*Bruguiera gymnorhiza* and *Sonneratia alba*) leaf extracts [78], gelatin with *Ficus carica* L. extract [74], and gelatin/chitosan film blends with betel leaf ethanol extract [79].

The films incorporated with clove extract (CE) demonstrated a high reducing power that increased with higher concentrations of CE. A higher absorbance indicates excellent reducing power. Eugenol, the main antioxidant component found in clove extract, significantly enhances the reducing power of the extract through its ability to bind with redox-active metal ions. This binding is crucial, as redox-active metals, like iron and copper, can promote the generation of free radicals, which contribute to oxidative stress and the deterioration of food quality [18,73]. By chelating these metal ions, eugenol reduces their pro-oxidant influence, thereby stabilizing the food matrix and extending its shelf life. The multifunctional characteristics of eugenol, along with its natural source, make it an important ingredient for developing sustainable and effective antioxidant solutions in seafood products.

#### 2.7.2. Antibacterial Properties of the Films

The results of the antibacterial assay of the produced films against four different bacterial strains associated with foodborne diseases are summarized in Table 4. The control film exhibited no antibacterial properties. In contrast, the films with 0.3% and 0.7% CE displayed a significant difference when compared to the control film. As the concentration of the extract increased, the antibacterial effectiveness of the films improved as well, resulting in a marked difference between the films containing 0.3% and 0.7% CE (*p* < 0.001). The greatest antibacterial activity was noted against *Staphylococcus aureus* and *Listeria monocytogenes*. In a similar study, Lv et al. [80] reported that clove bud extract exhibited antibacterial properties against *E. coli*, *S. aureus*, *P. aeruginosa*, and *L. monocytogenes*, with the most effective activity noted against *S. aureus*. Additionally, Sutrisno et al. [81] found that edible films incorporated with clove essential oil were capable of preventing the growth of *E. coli* and *S. aureus*. Furthermore, gelatin/guar gum films incorporated with grape seed oil demonstrated similar effectiveness against *S. aureus* [29].

The diameter of the inhibitory zone indicates the antibacterial potential of the extract [82]. The antibacterial compounds are categorized based on the inhibition zone into four different groups: very strong (diameters > 20 mm), strong (10–20 mm), moderate (5–10 mm), and weak (<5 mm) [14]. Our findings indicated that the antibacterial activity of the film incorporated with clove bud extract demonstrated moderate effectiveness, showing efficacy against the Gram-positive bacteria *S. aureus* and *L. monocytogenes* compared to the Gram-negative bacteria *E. coli* and *P. aeruginosa*. Likewise, other studies have noted that gelatin films containing *Ficus carica* L. extract, gelatin-based films with Ginkgo biloba extract, and gelatin/guar gum films containing grape seed oil also exhibited similar properties [14,29,83].

The pronounced antimicrobial efficacy of phenolic compounds is attributed to their capacity to induce structural and functional damage to the bacterial cytoplasmic membrane, as well as to disrupt intracellular metabolic processes [84]. As mentioned in the above sections, eugenol, the predominant phenolic compound in clove bud extract, is present in high concentrations, which enhances the extract’s potential antibacterial and antioxidant properties. Research has shown that eugenol compromises the integrity of the bacterial cell membrane, increasing its non-specific permeability [85]. Furthermore, due to its lipophilic characteristics, eugenol can disrupt cellular structures by interacting with the lipopolysaccharide layer of the bacterial cell membrane. This interaction leads to the leakage of intracellular substances, ultimately resulting in cell death [86]. Additionally, it has been suggested that the hydroxyl groups found in polyphenols act as proton exchangers, contributing to the destabilization of bacterial cell membranes. This destabilization can reduce the pH gradient and disrupt the membrane’s ion permeability, which subsequently affects electron transport and proton motive forces. As a result, essential components, such as ATP, necessary for bacterial metabolism, are released, leading to the elimination of bacterial cells [86,87].

In this study, the maximum concentration of the bud extract was 0.7% and the antibacterial activity was moderate, but if concentrations higher than 0.7% are used for the incorporation of the fish gelatin, the antibacterial properties of the films may be enhanced. However, this idea need more study.

## 3. Conclusions

In this research, various concentrations of clove bud extract (CE) were integrated into gelatin films derived from Southern meagre fish (*Argyrosomus hololepidotus*) to develop films suitable for seafood packaging. The study explored the impact of CE on the films’ properties. The incorporation of CE into the gelatin films significantly improved their water vapor barrier capabilities, resulting in enhanced water resistance. Notably, a concentration of 0.7% CE yielded films with a smooth surface, a dense cross-section, and superior seal strength. Additionally, the thermal properties of the gelatin films with CE showed increased thermal stability, along with a rise in the glass transition temperature (T_g_). Importantly, the antioxidant capacity of the fish gelatin films was enhanced through the use of CE. These findings suggest that biodegradable gelatin films from Southern meagre fish, enriched with CE, hold considerable promise as antioxidant active packaging materials. However, the antibacterial effectiveness of the films containing CE was found to be moderate. These films may also serve to extend the shelf life of seafood products, as indicated by preservation studies conducted on these items. Nevertheless, further research is necessary to assess their performance across various types of seafood products.

## 4. Materials and Methods

### 4.1. Materials

Clove buds were purchased from the domestic market in Tehran, Iran, while meagre fish skin was obtained from the fishmonger market in Chabahar, Iran. Glycerol, solvents, and DPPH were sourced from the Sigma-Aldrich (Tehran, Iran). All solvents and chemicals used in this study were of analytical grade.

### 4.2. Fish Skin Preparation

Meagre fish (*Argyrosomus hololepidotus*) skin was prepared from fresh fish, stored on ice at a ratio of 1:2 (*w*/*w*), and transferred to the Biotechnology Laboratory of the Marine Sciences Faculty at CM University (Chabahar, Iran). The skins were washed and descaled, and the meat was removed from the surface using a sharp knife (Zanjan, Iran). The skins were then cut into 1 cm × 1 cm pieces. The prepared skins were stored at −20 °C before gelatin preparation.

### 4.3. Fish Skin Gelatin Preparation

**Figure 5:**
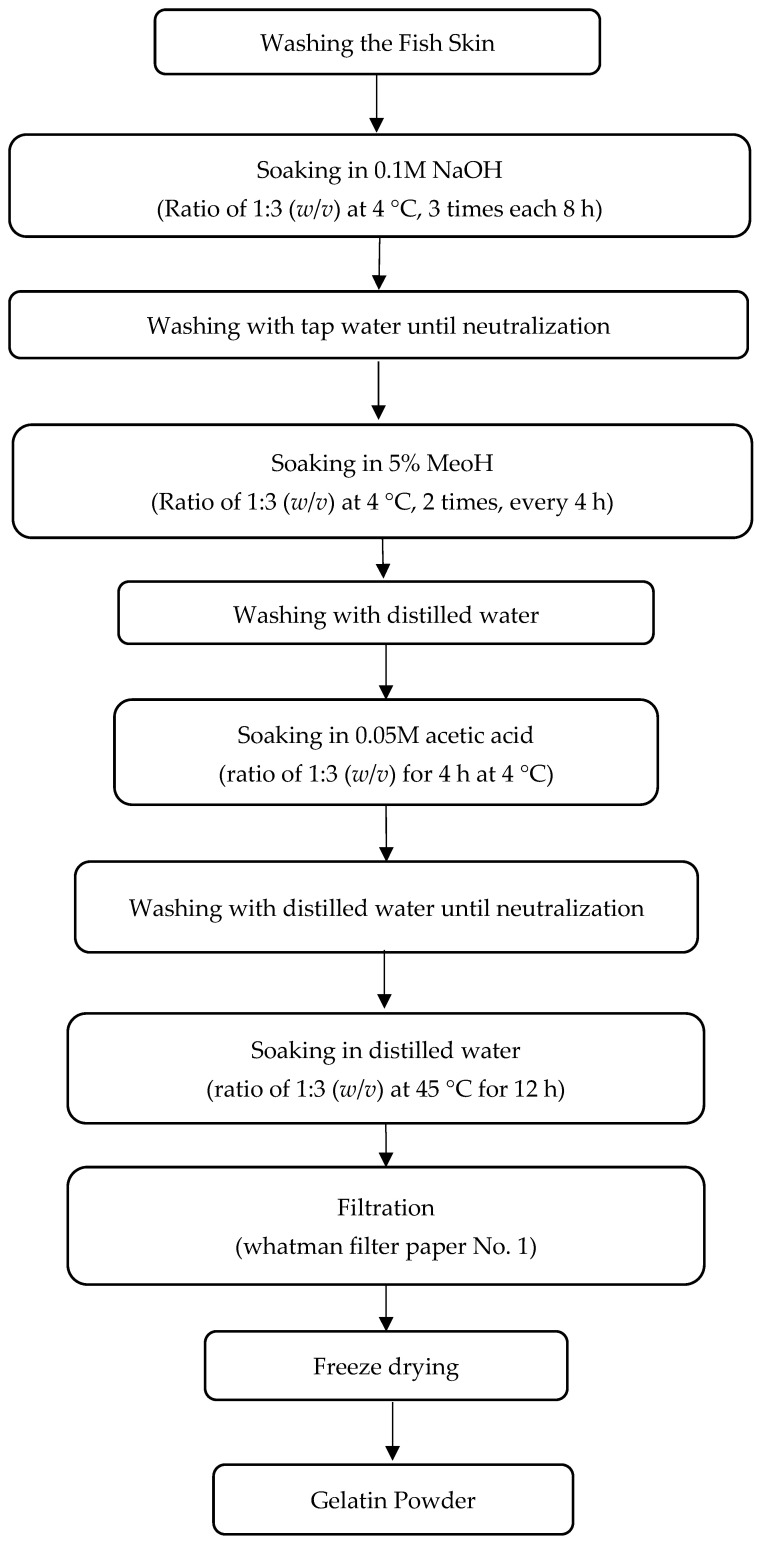
Schematic representation for the preparation of gelatin powder from meagre fish skin.

The process of gelatin extraction followed the method outlined by Wu et al. [31] with some changes. In brief, fish skins were soaked in a 0.1 M NaOH solution at a 1:3 (*w*/*v*) ratio at 4 °C while being stirred gently. The solution was changed every 8 h (three cycles) to eliminate non-collagenous materials. After the alkaline treatment, the skins were rinsed with tap water until the wash water reached a neutral or slightly basic pH (7–7.5). Next, the skins were immersed in a 5% methanol solution at the same skin-to-solution ratio (1:3 *w*/*v*) at 4 °C, with the solution replaced every 4 h for two cycles to remove fats. The skins were then washed with distilled water and treated with 0.05 M acetic acid at a 1:3 (*w*/*v*) ratio for 4 h at 4 °C while stirring gently to allow the collagen to swell. The treated skins were rinsed again as previously described. The swollen materials were soaked in distilled water at a 1:3 (*w*/*v*) ratio at 45 °C for about 12 h with continuous stirring. The mixture was then filtered using Whatman paper No. 1, and freeze-dried with a freeze dryer (Jal Teb, Tehran, Iran) to produce gelatin powder (Figure 5).

### 4.4. Preparation of Clove Bud Extract (CE)

Clove buds were processed using an electric grinder (Fuma, FU-341, Fuma Japan Corporation, Deira, UAE) to create a fine powder. Subsequently, 10 g of this powder were added to 100 mL of hot water (100 °C) and incubated in a water bath for 10 min. Once it cooled to room temperature, the extract was filtered using Whatman No. 1 filter paper. The final solution was then freeze-dried with a freeze dryer (Jal Teb, Tehran, Iran), and the resulting dry material known as ‘CE’.

### 4.5. Film Preparation

Film solutions were created by dissolving 4% (*w*/*v*) fish gelatin powder in distilled water under magnetic stirring at 900 rpm for 60 min at 45 °C. Glycerol was then added at a fixed amount of 25% (*v*/*v* relative to the fish gelatin), and stirring continued for an additional 15 min at 45 °C. Then, the mixture was centrifuged at 4000 × *g* for about 15 min to remove any impurities. CE powder was added to the film solutions at 0.3 and 0.7% (*w*/*v*). The solutions were continuously stirred at 500 rpm for an additional 15 min. A solution was made without any CE powder as a control. The film solutions were poured into petri dishes and left to air dry at 24 °C for 24 h. All films were stored in sealed plastic bags and refrigerated prior to further analysis [12,31].

### 4.6. Film Thickness

A digital micrometer was utilized to measure the thickness of the film at five different locations with an accuracy of ±0.001 mm. The average value was determined to represent the thickness of the edible film [12].

### 4.7. Solubility of the Film

The solubility of the films was measured following the method outlined by Gómez-Estaca et al. [45]. Dried films with dimensions of 1 × 3 cm^2^ were weighed and subsequently submerged in 15 mL of distilled water for 24 h while being continuously agitated at 22 °C. After this period, the undissolved films were dried at 105 °C for 24 h. The weight of the dissolved dry matter was determined using the following Equation (1):

Solubility (%) = [(W_i_ − W_f_)/W_i_] × 100 (1)

where W_i_ represents the initial weight of the dry film and W_f_ is the final weight of the undissolved dry film.

### 4.8. Water Vapor Permeability (WVP)

The water vapor permeability (WVP) was determined gravimetrically using the wet bottle method, following Wu et al. [31] with some adjustments. This approach aligns with the ASTM D1653-13 standard [88]. A bottle with a 5 cm inner diameter was filled with distilled water (of 6 mm height) and sealed with the film using parafilm to fix the film. After weighing, the sealed bottles were kept in a desiccator containing silica gel under controlled humidity and temperature conditions (30 °C for 10 h at 50% relative humidity). The weight of the sealed bottles was measured at one-hour intervals using a digital balance. WVP was calculated using the following Equation (2):

WVP (g m⁻^1^ Pa⁻^1^ s⁻^1^) = (W × X)/(A × t × ΔP) (2)

where W denotes the change in weight of the bottle (g), X refers to the thickness of the film (m), A represents the area of the exposed film (m^2^), t indicates the time (s), and ΔP signifies the difference in partial vapor pressure across the film (Pa).

### 4.9. Light Transmittance (T%) and Transparency Value

The films were cut into 2 cm × 1 cm strips and stuck to the inside wall of a quartz cuvette. The UV–visible spectra of the gelatin films were recorded in the range of 200 to 800 nm using a UV–visible spectrophotometer (160 A, Shimadzu, Japon). Transparency values were measured at 600 nm and normalized based on the film thickness (x), as in the following Equation (3) [45]:

Transparency = –log T600/X(3)

where Abs 600 is the absorbance at 600 nm

### 4.10. Scanning Electron Microscopy (SEM)

A scanning electron microscope (Hitachi S-4800; Tokyo, Japan) was used to analyze the morphology of the surface and cross-section of the film samples through imaging [89]. The samples were fixed on stubs and coated with gold using a sputter coater. Photographs were taken at a voltage of 15 kV.

### 4.11. Fourier Transform Infrared (FTIR) Spectroscopy

The structural relations between gelatin films and CE were examined using an FTIR spectrometer (PerkinElmer FT-IR, Spectrum-RXI, Shelton, USA). Approximately 30 mg of each film was compressed with 100 mg of KBr at an isostatic pressure of 150 MPa to create a pellet with a thickness of 200–300 μm. Spectra were assayed in the range of 400 to 4000 cm⁻^1^, with a resolution of 4 cm⁻^1^ and 32 scans [90].

### 4.12. Differential Scanning Calorimetry (DSC)

The thermal analysis of the films was conducted using a differential scanning calorimetry (DSC) device. A power compensation DSC (Mettler Toledo DSC 2 STAR System) was employed for the analysis. All experiments were conducted under a dry nitrogen atmosphere (50 mL N_2_/min) to prevent oxidation. Samples weighing 10 mg were tightly packed into aluminum pans. An empty pan used as the reference. The pans were heated in the range of 30–250 °C at a rate of 10 °C/min for thermal properties analysis. The onset temperature (T_onset_) and peak melting temperature (T_m_) were measured [91,92]. The glass transition temperature (T_g_) value was determined through software calculations utilizing DSC.

### 4.13. Bioactivity Assay

#### 4.13.1. Antioxidative Effect of the Films

##### Film Preparation for Antioxidant Assay

A 20 mg film was dissolved in 10 mL of 50% ethanol and then centrifuged at 6000× *g* for 15 min. The resulting supernatant was utilized for subsequent analysis.

##### DPPH Free Radical Scavenging Assay

The radical scavenging capacity was evaluated according to the method described by Cai et al. [93] with some modifications. In brief, 2 mL of the film solution was combined with 2 mL of a DPPH solution (2 mg in 50 mL of 95% ethanol) and incubated in a dark cabinet (30 min). The absorbance was measured using a spectrophotometer (517 nm) (Unico, 2100S, Shanghai, China) (A_g_). Additionally, 2 mL of the film supernatant was mixed with 2 mL of 50% ethanol and measured at 517 nm (A_c_). For the control, 1 mL of distilled water was added to 1 mL of ethanol, which was then mixed with 2 mL of the DPPH ethanol solution, incubated for 30 min, and measured at 517 nm (A_o_). All measurements were conducted in triplicate, with ascorbic acid used as the standard (0.02 mg/mL). The radical scavenging activity was calculated based on the following Equation (4):

Radical scavenging activity (%) = (1 − (A_g_ − A_c_)/A_o_) × 100. (4)

##### Reduction Power of the Film

The iron (III) reducing power of the films was measured using the method established by Oyaizu [94] with some modification. To summarize, 1 mL of the sample was mixed with 1 mL of 0.2 M phosphate buffer (pH 6.6) and 1 mL of a 1% potassium ferricyanide solution. This mixture was incubated at 50 °C for 20 min. After incubation, 1 mL of 10% (*w*/*v*) TCA was introduced, and the mixture was centrifuged at 10,000 × *g* for 10 min. Following this, 2 mL of deionized water and 0.4 mL of 0.1% ferric chloride were added to 2 mL of the supernatant. The absorbance was then measured at 700 nm after allowing a reaction time of 10 min. A higher absorbance value indicated the increased reducing power of the mixture.

#### 4.13.2. Antibacterial Effect

The disc diffusion method was employed to assess the antibacterial effects of the control and CE films, following the methodology outlined by Martucci et al. [95]. Four different foodborne pathogenic bacteria were utilized for the antibacterial assay: *Staphylococcus aureus* (ATCC 6538), *Listeria monocytogenes* (CECT 5873), *Pseudomonas aeruginosa* (ATCC 9027), and *Escherichia coli* (ATCC 8739). The lyophilized bacterial strains were purchased from the Iranian Pasture Institute. A volume of 15 µL of the extract solution was applied to blank discs, which were then placed on the surfaces of plates containing tryptic soy agar (TSA) or plate count agar (PCA). Prior to this, the plates were inoculated with approximately 107 colony-forming units per milliliter of the tested bacteria. Following inoculation, the plates were incubated at 37 °C for 24 to 36 h. The diameters of the inhibition zones were measured in triplicate.

### 4.14. Statistical Analysis

A one-way ANOVA was used to compare the treatments, and a Tukey test was employed to compare the mean values (*p* < 0.05). Statistical analyses were performed using Graph-Pad Prism 7, while Excel 2016 was utilized for figure preparation.

## Figures and Tables

**Table 1:** Thickness, water vapor permeability (WVP), and water solubility (WS) of fish gelatin films incorporated with clove bud extract (CE).

Film Type	Thickness (mm)	WS (%)	WVP (×10^−13^ g s^−1^m^−1^Pa^−1^)
Control without CE	0.26 ± 0.01 ^a^	89.20 ± 2.40 ^a^	1.68 ± 0.13 ^b^
Film incorporating 0.3% CE	0.24 ± 0.01 ^a^	84.44 ± 6.28 ^ab^	1.31 ± 0.21 ^b^
Film incorporating 0.7% CE	0.24 ± 0.01 ^a^	69.04 ± 3.37 ^b^	0.85 ± 0.10 ^a^

Values are expressed as mean ± standard deviation. Data with a different letters of a, b, in the same column indicate a significance difference at *p* < 0.05.

**Table 2:** Light transmittance (%) and transparency values of fish skin gelatin films incorporated with clove bud extracts (CE).

Film Type	Transmittance (nm)							
	**200**	**280**	**350**	**400**	**500**	**600**	**800**	**Transparency Values**
Control without CE	0.06	0.118	0.306	0.447	0.668	0.804	0.825	0.36 ± 0.014 ^a^
Film incorporating 0.3% CE	0.01	0.03	0.115	0.216	0.21	0.318	0.456	2.07 ± 0.086 ^b^
Film incorporating 0.7% CE	0.002	0.009	0.100	0.115	0.104	0.305	0.455	2.15 ± 0.089 ^b^

Transparency Values are expressed as mean ± standard deviation. Data with a different letters of a, b, in this column indicate a significance difference at *p* < 0.05.

**Table 3:** Glass transition temperature (T_g_), onset temperature (T_onest_), and T_m_ of fish skin gelatin films with clove bud extract (CE).

Film Type	T_g_	T_onset_	T_m1_	T_m2_
Control film without CE	51.04	45.62	124.65	134.30
Film incorporating 0.3% CE	57.12	53.30	124.19	134.20
Film incorporating 0.7% CE	58.80	47.35	141.92	171.83

**Table 4:** Antibacterial effect of fish skin gelatin films with clove bud extract (CE).

	Inhibition Zone (mm)			
Film Type	*Staphylococcus aureus*	*Pseudomonas aeruginosa*	*Escherichia coli*	*Listeria monocytogenes*
Control without CE	0 ^c^	0 ^c^	0 ^c^	0 ^c^
Film incorporating 0.3% CE	3.9 ± 0.10 ^b^	3.7 ± 0.36 ^b^	2.77 ± 0.30 ^b^	3.13 ± 0.13 ^b^
Film incorporating 0.7% CE	6.27 ± 0.42 ^a^	6.03 ± 0.25 ^a^	4.90 ± 0.62 ^a^	6.23 ± 0.15 ^a^

Note: The values are expressed as means ± sd (*n* = 3). Data with different letters of a, b, c, in each column show significant differences (*p* < 0.001).

## Data Availability

Data are contained within the article.
